# Advancing Dementia Research in Canada: Early Insights From a Scoping Review of Reviews

**DOI:** 10.1093/geroni/igaf122.4061

**Published:** 2025-12-31

**Authors:** Juanita-Dawne Bacsu, Kiana Mero, Alixe Menard, Megan E O’Connell, Jennifer Bethell, Sarah Fraser, Sheila Blackstock, Wendy Hulko

**Affiliations:** Thompson Rivers University, Kamloops, British Columbia, Canada; Thompson Rivers University, Kamloops, British Columbia, Canada; University of Ottawa, Ottawa, Ontario, Canada; University of Saskatchewan, Saskatoon, Saskatchewan, Canada; University Health Network, Toronto, Ontario, Canada; University of Ottawa, Ottawa, Ontario, Canada; Thompson Rivers University, Kamloops, British Columbia, Canada; Thompson Rivers University, Kamloops, British Columbia, Canada

## Abstract

Recently, a national dementia strategy was developed to advance the field of brain health and dementia in Canada. However, there is a paucity of information on the current research landscape and evidence available to advance this strategy. Drawing on findings from our recent scoping review of reviews, this late breaking presentation aims to: i) identify the landscape of Canadian-led reviews on brain health and dementia research; and 2) recognize knowledge gaps and areas of innovation to strengthen brain health and dementia research, policy, and practice. Drawing on Arksey and O’Malley’s framework, a comprehensive scoping review protocol was developed which guided our search of five databases (CINAHL, PubMed, PsycINFO, Scopus, and Web of Science). The literature was independently screened by two reviewers, extracted into a standardized table, and thematically analyzed. We found a total of 7860 initial articles, with 275 reviews included in our analysis. Findings were identified in three areas: 1) risk reduction strategies related to modifiable risks, lifestyle choices, and pharmacological factors; 2) enhancing therapies and finding a cure ranging from neuroimaging techniques to biomedical research; and 3) enhancing the quality of life of people living with dementia and care partners using a range of strategies to improve mental, social, and physical aspects. Although extensive reviews exist, significant knowledge gaps remain driven by factors such as limited participant diversity, inconsistent measures, and lack of targeted coordination, among other challenges. Findings from this study reveal vital gaps and research priorities that are essential to advancing Canada’s dementia strategy forward.

